# Portable dielectrophoresis for biology: ADEPT facilitates cell trapping, separation, and interactions

**DOI:** 10.1038/s41378-024-00654-z

**Published:** 2024-03-01

**Authors:** Lourdes Albina Nirupa Julius, Dora Akgül, Gowri Krishnan, Fabian Falk, Jan Korvink, Vlad Badilita

**Affiliations:** Department, Institute of Microstructure Technology, Karlsruhe Institute of Technology, Hermann-von-Helmholtz-Platz 1, Eggenstein-Leopoldshafen, 76344 Baden-Württemberg Germany

**Keywords:** Electrical and electronic engineering, Microfluidics

## Abstract

Dielectrophoresis is a powerful and well-established technique that allows label-free, non-invasive manipulation of cells and particles by leveraging their electrical properties. The practical implementation of the associated electronics and user interface in a biology laboratory, however, requires an engineering background, thus hindering the broader adoption of the technique. In order to address these challenges and to bridge the gap between biologists and the engineering skills required for the implementation of DEP platforms, we report here a custom-built, compact, universal electronic platform termed ADEPT (adaptable dielectrophoresis embedded platform tool) for use with a simple microfluidic chip containing six microelectrodes. The versatility of the open-source platform is ensured by a custom-developed graphical user interface that permits simple reconfiguration of the control signals to address a wide-range of specific applications: (i) precision positioning of the single bacterium/cell/particle in the micrometer range; (ii) viability-based separation by achieving a 94% efficiency in separating live and dead yeast; (iii) phenotype-based separation by achieving a 96% efficiency in separating yeast and *Bacillus subtilis*; (iv) cell–cell interactions by steering a phagocytosis process where a granulocyte engulfs *E. coli RGB-S* bacterium. Together, the set of experiments and the platform form a complete basis for a wide range of possible applications addressing various biological questions exploiting the plug-and-play design and the intuitive GUI of ADEPT.

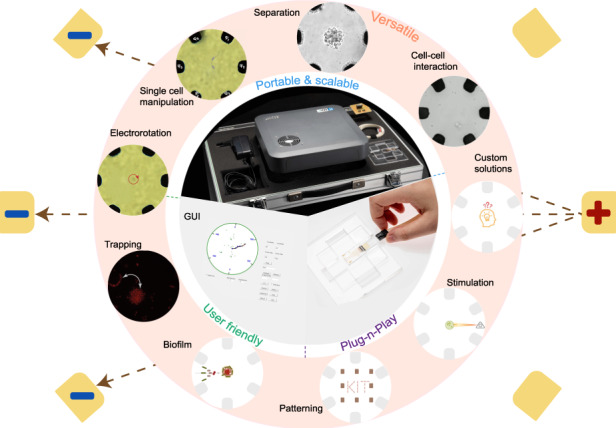

## Introduction

Microfluidics used in tandem with MEMS technologies has revolutionized cell biology by providing precise control and manipulation of cells, facilitating in-depth studies of cell behaviour under controlled conditions^1^. In the diverse areas of cell manipulation methodologies employed within microfluidics, various principles such as hydrodynamic, magnetic, optical and electrical have been exploited^2^. Among these methods, dielectrophoresis (DEP) stands out as a label-free, non-destructive and highly selective method^3–5^. In DEP, cells are manipulated based on their polarizability in a non-uniform electric field^6,7^. The precise cell positioning capabilities of DEP allow for selective separation of cells, manipulation and patterning of cells, as well as establishing controlled interactions among them^8,9^.

**Fig. 1:**
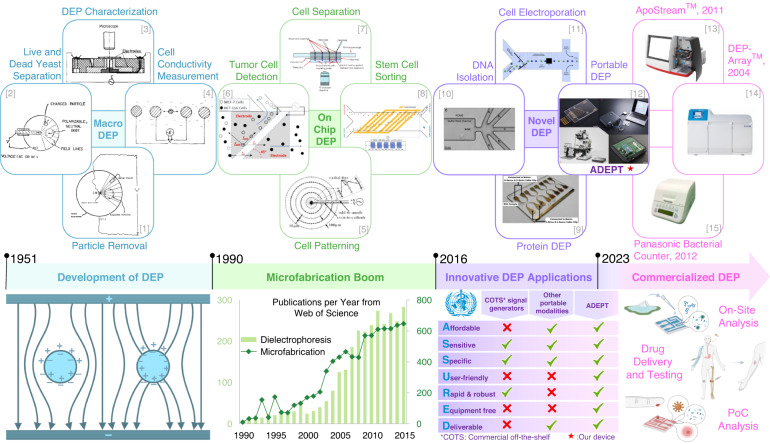
From initial development to commercialization. The evolution of the type of applications employing DEP technology. Panel [1] reproduced with permission from Pohl et al.^10^ (1951); copyright ⓒ 1951, American Institute of Physics. Panel [2] reproduced with permission from Pohl et al.^11^ (1966); copyright ⓒ 1966, The American Association for the Advancement of Science. Panel [3] reproduced with permission from Stoicheva et al.^13^ (1986); copyright ⓒ 1986, Wiley Online Library. Panel [4] reproduced with permission from Dimitrov et al.^12^ (1987); copyright ⓒ 1987, Elsevier. Panel [5] adapted with permission from Lin et al.^9^ (2006); copyright ⓒ 2006, Wiley Online Library. Panel [6] reproduced with permission from Alshareef et al.^16^ (2013); copyright ⓒ 2013, AIP Publishing. Panel [7] adapted from Lapizco et al.^15^ (2004); copyright ⓒ 2004, ACS Publications. Panel [8] reproduced with permission from Song et al.^19^ (2015); copyright ⓒ 2015, Royal Society of Chemistry. Panel [9] reproduced from Mohamad et al.^26^ (2017); copyright ⓒ 2017, AIP Publishing. Panel [10] reproduced with permission from Jones et al.^56^ (2017); copyright ⓒ 2017, ACS Publications. Panel [11] reproduced with permission from Punjiya et al.^27^ (2019); copyright ⓒ 2019, Nature Publishing Group UK London. Panel [12] reproduced with permission from Julius et al.^33^ (2023); copyright ⓒ 2023, Elsevier. Panel [13] adapted from Balasubramanian et al.^57^ (2017); copyright ⓒ 2017, Public Library of Science. Panel [14] adapted from Di Trapani et al.^31^ (2018); copyright ⓒ 2018, Wiley Online Library. Panel [15] adapted from Osako et al.^58^ (2021); copyright ⓒ 2021, MDPI

Pohl’s introduction of DEP in 1951 marked the initiation of a trajectory spanning seven decades, during which this technique underwent notable evolution. This narrative, depicted in Fig. 1, unfolds across four phases from the early development stage, progressing with the impact of microfabrication techniques that sparked a surge in DEP-based applications, transitioning into recent innovative approaches and ultimately culminating in its commercialization. Pohl initially used DEP to manipulate suspended particles in polymer solutions^10^. It was not until 1966 that Pohl and Hawk conducted experiments involving cells^11^. They employed a simple setup with electrodes and noticed that specific electrical signals attracted live cells to the electrodes. This laid the foundation for subsequent research, which included the characterization of dielectric properties of cells^12^ and resulting behaviours, including phenomena such as electrorotation and positive (pDEP) and negative (nDEP) DEP^13^.

Following this early development phase, the fusion of DEP technology with the emerging advancements in microfabrication techniques in the 1990s led to the creation of on-chip-DEP^14^. This combination led to a proliferation of DEP applications, expanding beyond live and dead cells to include a diverse array of cell types from bacterial to mammalian^3,15^ utilizing DEP in their detection^16,17^, sorting based on diverse characteristics such as differentiation stages^18,19^, and patterning^9^. For example, Jiang et al.^20^ developed a microfluidic cell separation system combining hydrophoresis and DEP for continuous high-throughput cell separation. The hydrophoresis module focuses cells into two streams, while the DEP module achieves cell separation, which was demonstrated successfully on murine neural stem cells. Ho et al.^21^ designed a microfluidic chip employing positive dielectrophoresis to pattern liver cells. The chip effectively traps liver cells, forming radial pearl-chain patterns and enabling simultaneous patterning of a substantial cell population. A 3D-printed microfluidic bioreactor was developed by Csapai et al.^22^ incorporating copper electrodes to harness DEP for initiating and cultivating biofilms. Their study showcased the preferential formation of biofilms by diverse bacterial strains in the high electric field region. These advancements indicate the growing popularity of DEP, inviting researchers to explore its application in increasingly novel contexts.

Upon the establishment of on-chip-DEP, the potential of DEP has surged with remarkable strides in innovative approaches (Phase 3)^23,24^. This progress exceeded the manipulation of cells and extended to the handling of cell-derived molecules such as DNA^25^ and proteins^26^, with the electric field being employed for tasks such as electroporation and transfection of cells with genetic material^27^.

Throughout all these stages, several attempts have been made to develop commercial products using DEP technology (see Fig. 1). For example, ApoStream^™^^28^ was designed specifically for isolating tumour cells from blood samples, while microarray devices developed by Biological Dynamics^29^ are aimed at isolating nucleic acids from the blood. The Panasonic bacterial counter^30^ utilizes DEP to determine bacteria concentration by measuring changes in electrical impedance, while the Silicon Biosystems DEPArray^™^^31^ employs DEP “field cages” to manipulate and isolate cells for further characterization. These devices excel in precise cell manipulation and effectively overcome the limitations associated with traditional cell manipulation methods by eliminating the need for extensive sample preparation and labeling. However, despite the advantages these commercial systems offer, they have not achieved widespread adoption^32^. This can be attributed to factors such as a lack of user-friendliness and a narrow focus on a specific application rather than addressing the broader need for a universally applicable device that can be easily integrated into any laboratory. Consequently, the continued dependence on bulky and complex signal generation equipment in laboratories has impeded DEP from broadening further than DEP-focused research groups, thereby hindering its deeper penetration into various branches of research.

To aid in this pursuit, we have developed and previously reported ADEPT^33^, the adaptable dielectrophoresis embedded platform tool, a portable, user-friendly, universal tool that meets the WHO Assured criteria^34^. This system harbours the potential to promote a wider and more rigorous use of DEP, a paradigm shift comparable to how the easy accessibility of micro-controllers revolutionized the embedded programming community and has been adopted universally to solve global problems, thereby democratizing computing and improving productivity. In contrast to commercial setups that lack in application flexibility and conventional systems that are often bulky and intricate, thus deterring researchers without an engineering background, ADEPT stands out as a versatile tool with a compact plug-and-play configuration. Complementing the robust hardware, the simple GUI ensures that biologists who possess the necessary domain-specific knowledge can define and implement biologically significant applications using DEP in their experiments. The assembly is completed by integrating a DEP chip with six microelectrodes arranged in a circular fashion, each independently controllable through its six signal generators (with the potential to easily scale up to twelve). The introduction of an additional pair of electrodes in the form of a circular arrangement, as opposed to the common quadrupole electrodes^35,36^, gives more functionality, such as high precision positional trapping and relocation of cells to a target position and patterning, alongside separation and electrorotation already achievable with other systems. To the best of our knowledge, this is the first time a portable, user-friendly DEP system with varied functionalities has been demonstrated in a single device.

This work demonstrates the adaptability and versatility of ADEPT by employing it in standard DEP procedures, such as viability and phenotype-based cell separation and takes a progressive step by exploring cellular interactions using the same microelectrode chip, which involves trapping *Escherichia coli* and granulocytes in proximity in order to analyse the phagocytosis process. Thus, with a single microelectrode chip design, various applications are demonstrated, and this versatility can be expanded to encompass many more applications, including patterning, biofilm formation, and cell stimulation. The purpose of these experimental demonstrations is to foster the widespread adoption of ADEPT among researchers spanning a diverse range of disciplines.

A detailed explanation of the electronics, experimental setup, cell sample preparation, experimental procedure, and image processing techniques employed in the study are provided in the Materials and Methods section. In the results and discussion section, we present the outcomes of separation and interaction experiments. Overall, ADEPT’s ease of use and versatility distinguishes it from the commercially available devices as well as conventional laboratory DEP setups, as a ubiquitous research tool. With its potential for comprehensive cellular analysis and applicability across various fields, ADEPT holds promise for exploring and expanding the potential applications of DEP technology in both academic and commercial settings.

## Results and discussion

### Cell experiments

**Fig. 2:**
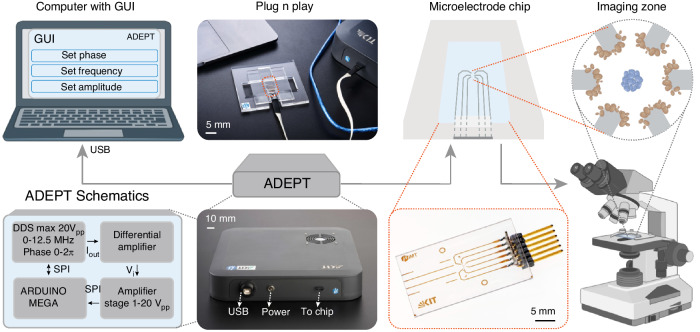
Schematic diagram of the experimental setup for DEP-based separation experiments—Adaptable Dielectrophoresis Embedded Platform Tool (ADEPT). ADEPT receives input from the computer hosting the GUI to input the desired excitation signals. Utilising the plug-and-play connections, ADEPT subsequently generates and transmits the signal to the chip, positioned on a holder beneath the microscope to visualise the cells within the imaging zone, i.e., the trapping zone encircled by the electrodes

All results were obtained using the experimental setup consisting of a custom-developed micro-electrode chip plugged into ADEPT and imaged using a microscopic setup as shown in Fig. 2. ADEPT consists of signal generator electronics which supply the control signals to activate the six electrodes independently. The control signals can be varied in frequency, phase, and amplitude by the user using a custom-developed graphical user interface (GUI). ADEPT also has the capability to select either a DC voltage (up to 5 V), ground (0 V), or a beat signal (mix of two frequencies) to activate each of the six electrodes instead of a single frequency signal. This is realized by a hardware multiplexer within ADEPT, which is also controlled through the GUI.

We conduct two types of experiments routinely performed using DEP with our module: 1) cell separation studies based on viability demonstrated using stained live and dead yeast and, based on cell phenotype using *B. subtilis* and yeast, and 2) the interaction between two different species by demonstrating the phagocytosis of *E. coli RGB-S* (Red-Green-Blue-Stress) reporter strain by HL-60 derived granulocytes.

#### Viability and phenotype-based separation

In addressing the need for a user-friendly DEP device, we show the ability of ADEPT to achieve high efficiency in cell separation.

**Fig. 3:**
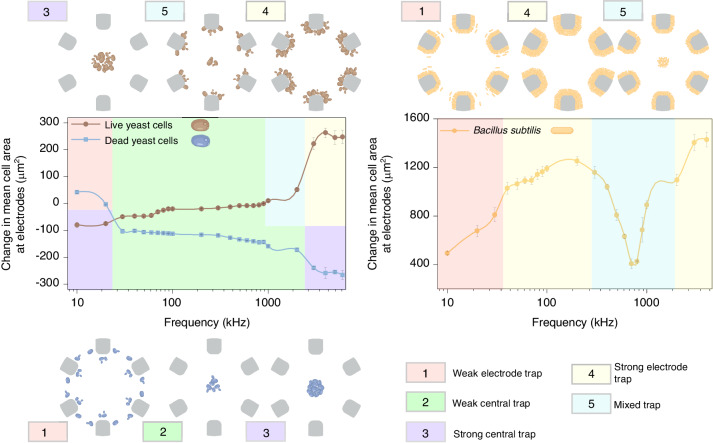
DEP trapping zone characterization of live and dead yeast and *B*. *subtilis*. A frequency sweep ranging from 10 kHz to 6 MHz was performed to assess the alteration in mean cell area at the electrodes of both live and dead yeast (displayed in the left graph), as well as *B. subtilis* (featured in the right graph), in order to investigate trapping behaviours across various frequencies. Accompanying illustrations surrounding the graphs facilitate comprehension of the trapping behaviour and subsequent positioning of the different cell types depending on the operating frequency

The separation of cells is based on the difference in the frequency-dependent behaviour of different cell types. As the operating frequency is varied, cells switch from exhibiting positive dielectrophoresis (pDEP) (i.e., movement towards the electrodes) to negative dielectrophoresis (nDEP) behaviour (i.e., movement away from the electrodes). The cross-over frequency is defined as the point at which the cells switch from nDEP to pDEP or vice versa, and certain cell types can have multiple such cross-over frequencies. Fig. 3 shows the frequency-dependent behaviour of the live and dead yeast and *B. subtilis* in the custom microchip with a circular arrangement of the microelectrodes. The frequency-dependent behaviour was carried out separately for each cell type. The y-axis of the plots in Fig. 3 shows the change in mean cell area at the electrodes. This was chosen as the metric since some cell types, such as the *B. subtilis* tend to attach to the device irreversibly. The change in mean cell area at the electrodes helps to consider only the cells actively moving to the electrodes because of the applied electric fields. The numbered colour boxes depict five different cell trap patterns, and the frequency ranges over which the cells undergo these patterns are highlighted in the plot by a similar colour to that in the numbered colour boxes. For instance, the strong central trap at the middle of the circular area of the six electrodes due to a strong nDEP behaviour of the cells is shown in the box numbered “3”. This trap pattern for the live and dead yeast happens in different frequency ranges, i.e., 5-15 kHz to well over 2 MHz for the live and dead cells, respectively. The other cell trap patterns include weak electrode trap, central trap, electrode trap and mixed trap. The threshold values of the change in the mean area of the trapped cells over frequency which defines these trap regions are mentioned in the methods sections. Mixed trapping occurs when cells of a particular cell type are trapped both in the center and at the electrodes at a given frequency. The occurrence of mixed trapping behaviour observed in both live yeast and *B. subtilis* within certain frequency ranges in our system can be attributed to the inherent cellular heterogeneity within populations of individual species^37,38^. Microorganisms at different cell cycle stages exhibit different morphologies and surface properties^39,40^. In addition, nutrient availability in the microenvironment can influence cell wall composition and surface properties and affect cellular polarity^41,42^. Changes in nutrient availability can also affect the localization of proteins involved in cell polarization, leading to changes in cell morphology^43^. Length variations within isogenic *B. subtilis* populations^44^ have been used to isolate subgroups with similar lengths. These inherent non-uniformities in cellular properties contribute to variations in trapping behaviour within the dielectrophoresis system, as individual cells respond differently to negative and positive DEP forces due to their dielectric properties.

ADEPT can vary both the frequency and phase shift between the control signals applied to each electrode, which leads to trapping cells at different positions within the circle enclosed by the electrodes (imaging zone). Supplementary Fig. S1 shows the experimental data on how the cell trap position changes when the phase of the sixth electrode is varied while the other five electrodes are set to fixed phase values. The plot also shows the additional points (shown in dark red) that can be targeted as a result of the circular symmetry of the microelectrode chip. A more rigorous study of the phase-controlled positional trapping is reported in our previous work^33^ where the standard deviations of the x-axis and y-axis coordinates of the trap positions are 1.5 *μ*m and 2.3 *μ*m, respectively. The phase positioning capability of ADEPT is used to further increase the separation between the two cell types.

**Fig. 4:**
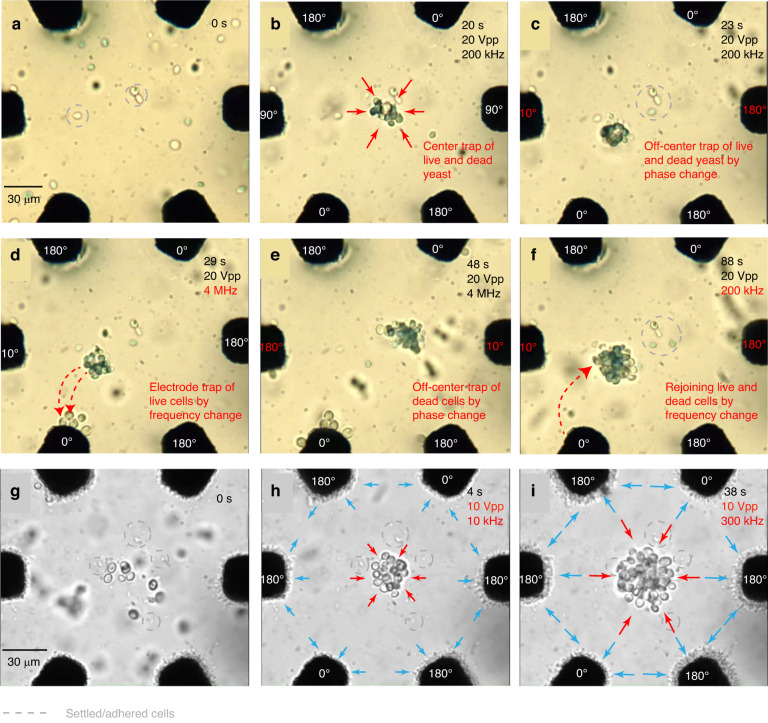
Viability- and phenotype-based separation. **a**–**f** Separation process of live (transparent) and dead (blue). The dead yeast takes up the methylene blue stain. **g**–**i** Separation process of live yeast and *B. subtilis*. The red and blue arrows denote the accumulation of the yeast and *B. subtilis*, respectively. The red dotted arrows indicate the transition of the cells from one trap position to another when the operating frequency is switched. The settled cells are marked out using gray dotted circles. **a**, **g** Represent initial conditions without the application of an electric field. In (**b**), 20 s after the field is applied with an amplitude of 20 Vpp and a frequency of 20 kHz, both live and dead yeast are trapped at the center. In (**c**), the phase combination is altered to move cells closer to the electrodes. In (**d**), the frequency is increased to 4 MHz, resulting in live cells being attracted to the electrodes, separating from the live-dead mixture. In (**e**), the dead cell cluster is moved away from live cells by adjusting the phase combination. **f** Demonstrates a frequency change back to 200 kHz to remix live and dead cells. In (**h**), *B. subtilis* and live yeast are depicted, with *B. subtilis* trapped at the electrodes and yeast at the center with field parameters of 10 Vpp amplitude and 10 kHz frequency. In (**i**), the frequency is increased to 300 kHz to attract more *B. subtilis* to the electrodes

For the separation of live and dead based on their viability, a signal with a frequency of 200 kHz and an amplitude of about 10 *V*_*pp*_ was applied to each of the six electrodes. The electrodes were given phase values of (0°, 90°, 180°, 0°, 90°, 180°) from electrodes 1 to 6, respectively, to initially trap both live and dead cells at the center (Fig. 4b). The electric field (E-field) distribution within the imaging zone is a superposition of the fields generated by the individual electrodes and results in low field strength at certain points within the imaging zone (in this case, the center) where the trapping happens. This low E-field trap location can be tuned by applying different phase combinations to the six electrodes. The trapped cells were then moved to a trap position between electrodes 4 and 5 by changing the phase sequence to (0°, 180°, 180°, 0°, 10°, 180°) (Fig. 4c). This ensures that the cells experience a stronger force during the separation process. The frequency and amplitude of the signal were maintained as before. To separate live and dead cells, the frequency and amplitude of the signal were changed to 4 MHz and 20 *V*_*pp*_, respectively (Fig. 4d), while maintaining the phase values. By changing the phase sequence (0°, 10°, 180°, 0°, 180°, 180°) (Fig. 4e), the position of the dead cells was shifted to a location opposite to that of the live cells within the imaging zone. The frequency and amplitude of the signal were kept constant to avoid detachment of the live cells from the electrodes. Both separation processes are depicted in Fig. 4a–f, illustrating the different steps involved in the separation procedure (see supplementary Video SI_video_live_dead_yeast). This method separated live yeast from dead yeast, achieving a separation efficiency of 94%. The separation efficiency was calculated using image processing techniques, which were described in the methods section of this paper, and an additional flowchart of the procedure is added in the supplementary information (Supplementary Fig. S7).

To perform the separation of different cell types, a mixed sample of *B. subtilis* and yeast was used, and the results are shown in Fig. 4g–i (see supplementary Video SI_video_bacillus_yeast_separation). The yeast cells were center trapped by setting the signal frequency to 10 kHz, amplitude to 10 *V*_*pp*_, and phase sequence to (0°, 90°, 180°, 0°, 90°, 180°). Subsequently, the frequency was adjusted to 300 kHz and the amplitude to 20 *V*_*pp*_ at the same phase sequence to maintain the yeast in the center while simultaneously trapping a larger number of *B. subtilis* on the electrodes. Since increased amplitude induced cell rotation, the amplitude was adjusted back to 10 *V*_*pp*_ to stabilize the cells at their trap position. This set of signal parameters led to a progressive expansion of *B. subtilis* area on the electrodes and an accumulation of yeast cells in the central region, signifying successful trapping of *B. subtilis* on the electrodes and yeast in the center. As a final adjustment, the frequency of the signal was tuned to 10 kHz to enhance the trapping of more yeast at the center. During the preparation process, some of the *B. subtilis* attached to the chip surface at various locations on the chip, including the microelectrode area. As these cells were observed in the same location both at the beginning of the experiment and after separation, they were not considered as trapped cells when determining the separation efficiency. *B. subtilis* exhibited a tendency to become trapped at the electrodes. At a frequency of 300 kHz, where yeast cells were trapped in the center and *B. subtilis* were trapped at the electrodes, the separation efficiency is 96%. The results show that ADEPT can be used for routine separation experiments, similar to state-of-the-art devices, while offering the added advantage of precise positioning of separated cell groups within the same imaging zone. Furthermore, by simply changing the frequency, the separated populations can rapidly be brought back together and re-separated, facilitating the observation of mixed and isolated cell groups. Separation occurs immediately after signal application, with cells being visibly trapped at specific locations within 4 seconds. If required, integrating a microfluidic channel in the chip would easily enable the elution of the separated cell groups.

While the specified parameters in our study yielded successful results for the cell types and buffers used in our experiments, it remains crucial to tailor the approach to the specific requirements of the cells under study. Variables such as cell size, dielectric properties, and buffer conductivity can significantly affect cell separation and trapping results. Therefore, it is necessary to identify the specific phase sequences and range of frequencies and amplitudes to determine the optimal conditions for specific cells. Optimal trapping conditions can be determined by observing frequency-dependent responses and identifying crossover frequencies at which cells switch from nDEP to pDEP. In addition, it is essential to consider the geometry and alignment of the microelectrodes to ensure accurate and reliable positioning of the cells for different experimental contexts.

### Interaction between *E. coli RGB-S* and HL-60 derived granulocytes

DEP has emerged as a widely utilized method for cell separation, yet its full potential in studying intercellular interactions remains largely unexplored. Traditional methods for studying cell–cell interactions, such as co-culture assays and flow cytometry, often involve extensive sample preparation and may not fully replicate the in vivo environment. In contrast, DEP-based techniques offer a promising alternative, allowing for precise and non-invasive manipulation of cells to facilitate their proximity and enable the analysis of cell–cell interactions. Previous applications of DEP in cell interactions have mainly focused on studying the formation and organization of multicellular networks, such as the patterning of liver cells, neural networks, and biofilms. However, investigating interactions between different cell types requires a system capable of inducing controlled attraction between cells, despite the variability in their dielectric properties. One example is the DEParray developed by Abonnenc et al.^45^, which features an array of more than 10,000 spherical DEP cages capable of levitating and moving microspheres and cells, enabling interactions between macrophages and target cells. However, this system involves complex electrode designs that require specialized microfabrication skills and intricate electronics, limiting its accessibility.

To address such challenges, we propose the use of ADEPT as a simpler and more accessible method for studying cell–cell interactions. ADEPT allows for the trapping of cells of different species in close proximity, facilitating the occurrence and analysis of cellular interactions.

**Fig. 5:**
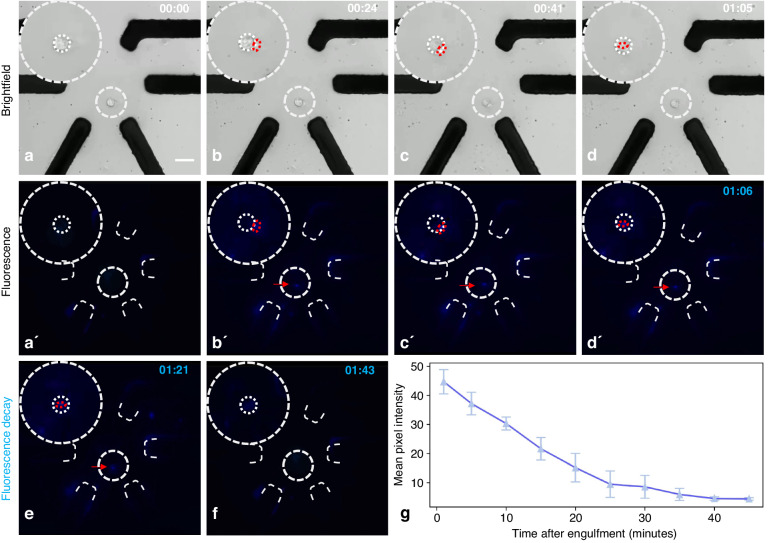
Phagocytosis process of an *E. coli* by a granulocyte. The top row illustrates brightfield images (**a**–**d**), while the second row displays corresponding fluorescence images (a'-d'). In (**a**), only the granulocyte is trapped in the imaging zone. **b** Both the granulocyte and the bacterium trapped together by applying a signal with a frequency of 200 kHz, an amplitude of 10 *V*_*pp*_, and a phase sequence of (0°, 180°, 180°, 0°, 10°, 180°). **c** Depicts the phagocytosis process, occurring approximately 37 minutes after trapping. **d** Demonstrates the bacterium engulfed by the granulocyte after 9 minutes since the initiation of phagocytosis. Additionally, (d') showcases the fluorescence image, highlighting the bacterium inside the granulocyte, marking the start of fluorescence decay. **e**, **f** Exhibit the decay of fluorescence over the next 30 minutes following complete engulfment. Finally, **g** portrays the decay of fluorescence over time, illustrated by the mean pixel intensity from four repeated measurements. The fluorescence intensity is halved after approximately 15 minutes and diminishes almost completely after approximately 40 minutes. To aid in visibility, a zoom image of the cell and its immediate surroundings was included in the top left corner of all images. In the zoom images, the area of the granulocyte was outlined with a white dashed circle, while the *E. coli* was marked with a red dashed circle and indicated by a red arrow. Furthermore, the electrodes were made visible in all fluorescence images using white dashed lines. To differentiate between the phagocytosis process and fluorescence decay, images were labeled with white text to indicate engulfment and blue text to represent the decay of bacterial fluorescence. Scale bar: 30 *μ*m

In this study, we investigate the interaction between the *E. coli RGB-S* reporter strain^46^ and HL-60 derived granulocytes using a combination of dielectrophoresis and fluorescence microscopy. By trapping the granulocytes and *E. coli* at the same location on the micro-electrode chip, we observed the phagocytosis process and observed the subsequent fluorescence decay within the granulocyte. The application of DEP increased the chances of granulocytes encountering *E. coli*. This overcame the limitations associated with random encounters between cells and allowed for controlled and reproducible studies of cell–cell interactions. Overall, this simple setup provides an easy-to-use system for studying complex cellular interactions. Fig. 5 portrays the engulfment process and the following decrease of the fluorescence intensity of the engulfed *E. coli RGB-S* within the granulocyte (See supplementary Video 3). Our results showed that once the cells were trapped together using DEP, the granulocytes initiated phagocytosis of the *E. coli* cells after approximately 40 minutes. The engulfment process took 10 minutes on average, highlighting the rapid and efficient phagocytic activity of granulocytes. After internalization, we observed a gradual decay of fluorescence within the granulocyte, which faded over a period of approximately 35 minutes and ultimately disappeared completely. This decay in fluorescence intensity indicates a gradual lysis of *E. coli*, due to enzymatic activity within the phagolysosomes of the granulocyte. In order to show that the lysis of *E. coli* was due to the granulocyte activity and was not caused by the electric field, a cell sample containing just *E. coli* suspended in RPMI medium was subjected to the electric field on the micro-electrode chip. As seen in the Supplementary Fig. S2, the *E. coli* were trapped in the center of the DEP chip, and the fluorescence was monitored for over 60 minutes, and the cells remained alive, as proven by the strong fluorescence signal. In the signal ranges in which we operate, it is important to consider and investigate the physiological effects of the electric field on the cells, such as electroporation, within the device. This consideration is vital for minimizing potential detrimental effects in future studies. Nevertheless, the observed capability of cells for phagocytosis suggests that the cellular physiology is not significantly compromised by the electric field, ensuring their viability.

These findings underscore the utility of DEP in enhancing the observation of cell–cell interactions. This approach provides a foundation for future investigations into the mechanisms underlying cell–cell interactions. Our study demonstrates the effectiveness of ADEPT in investigating the interaction between *E. coli* and granulocytes, laying the groundwork for the development of innovative techniques for studying complex cellular processes in various fields of research in a controlled and reproducible manner, as listed in Table 1.

**Table 1:** Applications of DEP in cell research, and potential impact of ADEPT

Application area	Examples from the literature	Enhancements offered by ADEPT
Separation	CMOS based separation of live and dead yeast (Matbaechi et al.)^51^. Characterization and separation of CTC by varying AC frequency (Alshareef et al.)^16^. iDEP based separation of live and dead *E.coli* samples (Lapizco et al.)^15^.	Continuous and rapid mixing and separating of cell mixtures, performed by varying frequencies. The separation distance is enhanced by tuning the phase combinations applied to the electrodes without the need for complex equipment. Using a phase combination that generates a double trap at two different locations^33^ enables the generation of two clusters of the total cell population, as required for quorum sensing studies.
Trapping	nDEP trapping combined with AC electrokinetics for electroporation (Punjiya et al.)^27^. Microfluidic DEP device with carbon electrodes for cell trapping (Puri et al.)^52^.	Trapping of single cells as well as multiple cells with a spatial precision of micrometers^33^. The cells can be dragged along a geometrical path simply by drawing the path on the canvas in the GUI.
Interactions	Induction of interactions between immune cells, tumor cells, and virus-infected cells in DEP “cages” (Abonnenc et al.)^45^. DEP pairing of endothelial cells and tumors to analyze interactions (Yin et al.)^5^.	Similar to the demonstration of the phagocytosis shown in this paper, ADEPT has the ability to reveal other known cellular interactions, such as parasitic dynamics. In addition, ADEPT can unveil interactions that remain undiscovered, determining whether certain cells engage in any symbiotic relationship. The impact of distance to initiate cell interactions is accomplished by tuning the distance between them.
Patterning^a^	DEP assisted liver cell patterning using microfluidics (Ho et al.)^21^. Neural network formation by utilizing nDEP of neural stem cells (Yu et al.)^53^. Insulator-based cell pairing on a microfluidic chip (Pendharkar et al.)^50^. Formation of artificial microbial consortia using DEP and flocculation(Csapai et al.)^48^.	Biofilms can be formed by moving cells along predefined paths and holding a cell at a desired position until the cell forms an irreversible attachment to the surface. Once the cells are stable at that location, a new group of cells can be trapped either at a different position (to obtain a specific pattern) or on top of the existing biofilm (to study the interaction). Different geometric cell patterns can be created with ADEPT by replacing the present chip with an alternate geometric electrode design.
Stimulation^a^	Assessment of bacterial and fungal cell viability following iDEP manipulation (LaLonde et al.)^54^. Study of the impact of a wide range of DEP field strengths on mammalian cells (Desai et al.)^55^.	Controlled external stimuli (mechanical/optical/chemical) can be induced on cells at precise positions on the cell body. By gradually and dynamically varying the stimuli, selective and specific stress conditions can be induced to influence their adaptive responses.

^a^Not presented here but could be easily implemented

## Conclusion and outlook

ADEPT, a user-friendly and versatile DEP device, successfully addresses the challenges of complex designs and specialized expertise. Through viability and phenotype-based separation, as well as the application of controlled attraction forces, ADEPT achieved high-efficiency cell separation and enabled the controlled and reproducible demonstration of proof-of-concept studies of cellular interactions. By providing precise positioning of separated cell groups within the same imaging zone, ADEPT enables simultaneous observation and analysis of intricate cellular processes, revealing cellular behaviour more precisely.

ADEPT proves to be a versatile and robust tool applicable to routine as well as novel scientific investigations. Researchers addressing various research questions can readily adopt ADEPT to perform cell patterning, isolate specific cell types from complex populations, and conduct electroporation experiments. Since the micro-electrode chip can be operated with standard culture media such as Luria Broth or RPMI 1640, the incorporation of a microfluidic channel into the chip allows for continuous cell culturing^47^, facilitating dynamic population studies and long-term interactions. Beyond routine applications, ADEPT opens up exciting possibilities for addressing challenging research questions. The study of biofilms^48^, composed of intricate networks of microorganisms, becomes feasible with precise control and manipulation facilitated by ADEPT. Additionally, the modality can enable colony formation studies and analysis of quorum sensing in microorganisms like *Aliivibrio fischeri*, shedding light on bacterial communication and collective behaviours in diverse ecosystems^49^. Another intriguing possible novel application of ADEPT is controlled cell fusion. By precisely positioning different cell types relative to each other, ADEPT can facilitate cell–cell interactions that lead to successful fusion^50^. This advancement holds tremendous potential in regenerative medicine, biotechnology, and the creation of artificial cells with unique properties.

## Methods

### Cell culture and preparation

In this study, cells of *Saccharomyces cerevisiae*, i.e., commercially available dried baker’s yeast, have been used. To prepare the stock solution, 300 mg dried baker’s yeast was suspended in 50 mL of a 2% glucose solution and incubated for 30 min at room temperature. The sample was transferred in equal parts to 2 mL microcentrifuge tubes. To obtain dead cells, a sample was heated to 100°C for 30 min in a dry bath block (LLG-uniTHERMIX, Lab Logistics Group GmbH, Meckenheim, Germany). Cells were pelleted by centrifugation at 3600 rpm for 5 min (Centrifuge 5810, Eppendorf SE, Hamburg, Germany) and resuspended in 500 *μ*L DEP buffer (10 mM TES buffer, 0.1 mM CaCl_2_, and 236 mM sucrose at a pH value of 8). The DEP buffer has a conductivity of 0.1 S/m.The conductivity measurements were carried out using the SevenCompact^TM^ S230 (Mettler-Toledo GmbH, Switzerland).

For separation, individual live and dead cell suspensions, as well as a 1:1 mixture of live and dead cells, were stained with methylene blue (MB) dye (Sigma-Aldrich, Merck KGaA, Darmstadt, Germany) in equal proportions. The samples were stained with MB for 2 min. The stained samples were further diluted with DEP buffer at a volume ratio of 1:10 for the separation experiments, due to the high cell concentration of the cell suspension.

To determine the viability of cells in DEP buffer, an overnight culture of yeast was diluted in a 1:100 ratio in DEP buffer and incubated at 30°C. Subsequently, 500 *μ*L of the cell sample was collected at regular intervals to measure the optical density at 600 nm (OD600), using the Cell Density Meter, Biowave, WPA CO8000 (Biochrom Ltd., Cambridge, UK). Along with the OD measurements, cell viability was assessed by methylene blue staining and counting the number of live and dead cells with a Neubauer improved counting chamber (Paul Marienfeld GmbH Co. KG, Lauda-Koenigshofen, Germany). Viable cell percentage was calculated by dividing the number of live cells by total cell number. The measurements were conducted every 30 min for the first 4 hours, then every hour, for a total duration of 9 hours. The results indicated that yeast cells maintained viability in DEP buffer for at least 6 hours. However, beyond this time frame, viability declined, coinciding with a plateau in the growth curve as seen in Supplementary Fig. S3. This investigation establishes the optimal time window for yeast experiments in DEP buffer.

#### *Bacillus subtilis*

DSM 402 cultures were grown at 30°C in Muller-Hinton-Agar plates (2 g/L beef extract, 17.5 g/L casein hydrolysate, 1.5 g/L starch, 17 g/L agar in DI water) for 24 h to reach saturation conditions. Prior to DEP experiments, cells were centrifuged at 5000 rpm for 15 min and resuspended in 1 mL DEP buffer.

#### *Escherichia coli*

The *RGB-S* strain was grown in Luria Broth (LB) 10 g/L tryptone, 5 g/L extract, 5 g/L NaCl, pH 7). The cell culture was incubated overnight at 37°C and subjected to 180 rpm in a shaking incubator (Thermoshake THL, C. Gerhardt GmbH & Co. KG, Königswinter, Germany). For DEP experiments, cell samples were centrifuged at 5000 rpm for 15 min and resuspended in 1 mL LB medium.

#### Opsonization of *E. coli*

The bacteria were cultured overnight, washed once with PBS, and resuspended in human plasma to a plasma concentration of 20%. The cells were incubated in human plasma at 37°C and subjected to 150 rpm for 60 min. The opsonized cells were washed once with PBS and resuspended in LB medium for further use.

### Cultivation and differentiation of HL-60 cells

The detailed preparation of the HL-60 cells is shown graphically in Supplementary Fig. S4. HL-60 cells were cultured in RPMI 1640 medium supplemented with 10% human plasma (pooled, blood-derived) and 1% penicillin/streptomycin. Cells were cultured at 37°C and 5% CO_2_ in a standard tissue culture incubator in 25 cm^2^ cell culture flasks with 0.2 *μ*m vent caps and passaged when cells reached a density of approximately 1 million cells/mL. To induce a neutrophil-like phenotype, cells were treated with 1.75% DMSO for four days. For experiments, differentiated cells were harvested by centrifugation at 360 × g for 8 min and adjusted to a density of 106 cells/mL in RPMI 1640. To isolate differentiated cells from the undifferentiated ones, 5 mL of the prepared sample was layered on 5 mL of Ficoll-Paque Plus solution in a centrifuge tube and centrifuged at 400 × g for 30 min. The differentiated cells were collected and suspended in RPMI 1640 supplemented with 2% human plasma, and the measured conductivity value of the medium was 1.5 S/m.

### Experimental setup

The components and operating principle of the signal generator unit, the design and manufacture of the microelectrode chip, as well as the graphical user interface have previously been described in detail^33^. This article aims to focus on the biological applications of ADEPT.

#### Micro-electrode chip preparation

The electrodes are fabricated on 500 *μ*m thick glass substrate coated with the Cr/Au (20 nm/60 nm) seed layer. A SU-8 mould for electroplating the gold electrodes was fabricated using a UV lithography process with the EVG®620 EV Group system. The SU-8 mould is 25 *μ*m thick, and the electroplated electrodes are 10 *μ*m high. After electroplating, the photoresist is stripped off using dry etching (R3T plasma etcher) and the seed layer is removed using a wet chemical etching process. The fabricated electrodes have a width of 30 *μ*m at the tips, and the imaging area enclosed by these electrode tips have a diameter of 100 *μ*m. A 6-pin header is directly soldered onto the electrical pads on the micro-electrode chip after cutting through holes in glass using a nanosecond laser (PIRANHA® ACSYS, Germany). The micro-electrode chips were treated with 2.5 mM Ethylenediaminetetraacetic acid (EDTA)(Sigma Aldrich, Missouri, USA) and O_2_ plasma treatment (R3T plasma etcher). Although these additional steps aided in preventing the irreversible adherence of the cells and washing away the settled cells after the experiments, they did not prevent the settlement of the cells during the experiments.

#### Setup

As shown in Fig. 2, the experimental setup features a microelectrode chip containing six gold microelectrodes, each with a pin connector. The electrodes are connected to a microcontroller through a commercially available cable, which is also linked to a computer equipped with a graphical user interface via a USB port. The chip is placed on a PMMA holder, and 10 *μ*L of the sample is pipetted directly onto the electrodes before being covered with a coverslip. In this work, we aim to use an open-channel architecture as it is a similar approach to a microscope slide, which biologists routinely use. However, collecting the sample can be easily implemented using a microfluidic approach. Supplementary Fig. 5 shows that a PDMS (Polydimethylsiloxane) channel mould fabricated using soft lithography is oxygen plasma bonded (100 W for 8 seconds) to the micro-electrode chip (glass with gold electrodes) and fitted with tubing. The chip holder is positioned under the optical microscope with 40x magnification (Leizt Ergolux 200, Leica Microsystems, Wetzlar, Germany). We also used the microscope (Nikon Ti-E, Nikon Corp., Tokyo, Japan) to record higher quality frames of the phenotype-based separation of the yeast and *B. subtilis*. The signal parameters were adjusted accordingly using the graphical user interface in order to trap cells at the desired locations.

### ADEPT

ADEPT has up to six identical electronic channels to control up to six electrodes independently. Conventional trapping systems use up to four electrodes. Here, we show that it is possible to achieve a higher spatial trapping resolution by using more electrodes. The capability of ADEPT to control more than six electrodes (the present design offers up to twelve channels) can be easily accomplished due to the scalable nature of the electronics design and is limited by the maximum current supplied by the power regulator. The core of each ADEPT electronic channel is the direct digital synthesizer (DDS) from Analog devices (AD9913) which generates the control signal with variable frequency and phase. The current output signal of the DDS is converted to a voltage output signal using a differential opamp configuration. An amplifier stage with digital potentiometers follows it to vary the amplitude of the control signal. An Arduino Mega (integrated into the ADEPT) is used to control the frequency (10 Hz-30 MHz with a step size of 0.058 Hz), phase (0° to 360° with a phase tuning resolution of 0.022°) and amplitude (0-20 Vpp) of the output signal. The frequency and phase are controlled directly at the DDS, and the amplitude of the control signal is varied by changing the resistance value of the digital potentiometer at the amplifier stage.

### Cell experiments

The viability-based separation experiments were imaged using an IDS camera (UI-3060CP-C-HQ R2, IDS Imaging Development Systems GmbH, Germany). The videos were recorded at a frame rate of 10.21 frames/s using the uEye Cockpit software (IDS Imaging Development Systems GmbH, Germany). Phenotype-based separation experiments, as well as the phagocytosis experiments, were recorded using a 40x/0.75 Nikon objective affixed to a Nikon Eclipse Microscope at a frame rate of 7 frames/s.

The separation mechanism relies on the frequency-dependent behaviour of cell types, transitioning from pDEP to nDEP as the frequency of the signal varies. To elucidate the trap behaviour of cells, we defined the trap strength at given frequency intervals based on the trapped cell area. The trap strength measures the change in mean cell area at the electrodes, calculated by subtracting the electrode cell area from the center trapped cell area. When quantifying the separation experiments, the area of the cells was taken as a metric since the constant phase combination applied kept the cells trapped at the center. Only when the crossover frequency is reached do the cells rapidly switch to the electrodes, where they immediately accumulate. Negative values of cell area arise due to this calculation method. If the cell area at the center is larger than at the electrodes, the area value is negative. This method allows us to visualize nDEP by using negative values and pDEP with positive values.

Due to the variations in trapped cell area among live yeast, dead yeast, and *B. subtilis* (which can be attributed to differences in their distinct electrical and morphological properties, resulting in individualized degrees of movement in response to the applied electric field), the trap strength for each cell type was determined individually. For *B. subtilis*, the weak/strong electrode trap threshold was 1000 *μ*m^2^. A cell area below this threshold was considered a weak trap, and over this threshold was a strong trap. Live yeast demonstrated weak center trapping within the area range of -50 *μ*m^2^ to 0, and weak electrode trapping for areas spanning 0 to 50 *μ*m^2^. Strong electrode trapping was assigned to cell areas exceeding 150 *μ*m^2^. In the instance of dead yeast, the threshold for weak electrode trapping was defined within the range of -50 *μ*m^2^ to 50 *μ*m^2^. For weak center trapping, the applicable range extended from -50 *μ*m^2^ to 250 *μ*m^2^, and for strong center trapping, it was below -250 *μ*m^2^. For microscopic images of cells displaying the above-mentioned behaviour, refer to Supplementary Fig. S6.

Quantification of the experiments was done by image processing. All experiments were conducted for a minimum of three replicates.

### Image processing

Quantification of the cell area of the trapped cells was done using ImageJ. For this purpose, the videos were segmented into frames, and the frames were converted to 8-bit format and inverted. Manual selection of the imaging zone followed, with the clearing of the region outside the selected imaging zone. Background subtraction was then applied to eliminate settled, adhered, or out-of-focus cells (which are unaffected by the electric field). Subsequently, the lower threshold was set at 168, and the upper threshold at 206.

To determine the change in cell area at the electrodes, an image was selected before applying the signal, and the cell area was measured. Then, the area of the cells was determined from an image after the stabilization of the cells at the trap position. The change in cell area was calculated by subtraction of the determined areas.

To calculate the separation efficiency, the area of cells within the mentioned threshold was measured. This provided the total cell area. Following this, the area of cells trapped at the center was marked out and measured, representing the area of the separated cell type (either dead yeast for viability-based separation or live yeast cells for phenotype-based separation). The separation efficiency was calculated by dividing the area of the separated cells trapped at the center by the total cell area. Viability-based separation efficiency was quantified 10 seconds after applying the signal to separate the cells, while phenotype-based separation was conducted 51 seconds after signal application, to ensure stable trapping at the quantification point. A comprehensive flowchart detailing each step of the efficiency quantification is provided, as examplified by the phenotype based separation in the supplementary information (see Supplementary Fig. S7).

Fluorescence decay quantification was accomplished by contouring the outline of the cell and measuring the pixel intensity within the contoured region for each frame of the video.

### Supplementary information

Supplementary Information

Updated SI_video_live_dead_yeast_separation

Updated SI_video_bacillus_yeast_separation

Viability based separation

Phenotype based separation

Phagocytosis

Portable dielectrophoresis for biology: ADEPT

## Data Availability

The data supporting this manuscript are available upon reasonable request.
