# Supplementary material for: Portable dielectrophoresis for biology: ADEPT facilitates cell trapping, separation, and interactions
Source: Microsyst Nanoeng. 2024 Mar 1;10:29. doi: 10.1038/s41378-024-00654-z (PMC10907756; doi:10.1038/s41378-024-00654-z)
Supplement: Supplementary file 1 — Supplementary Information [file 41378_2024_654_MOESM1_ESM.docx]

**Appendix A. Supplementary Information: Portable dielectrophoresis for biology: ADEPT facilitates cell trapping, separation, and interactions**


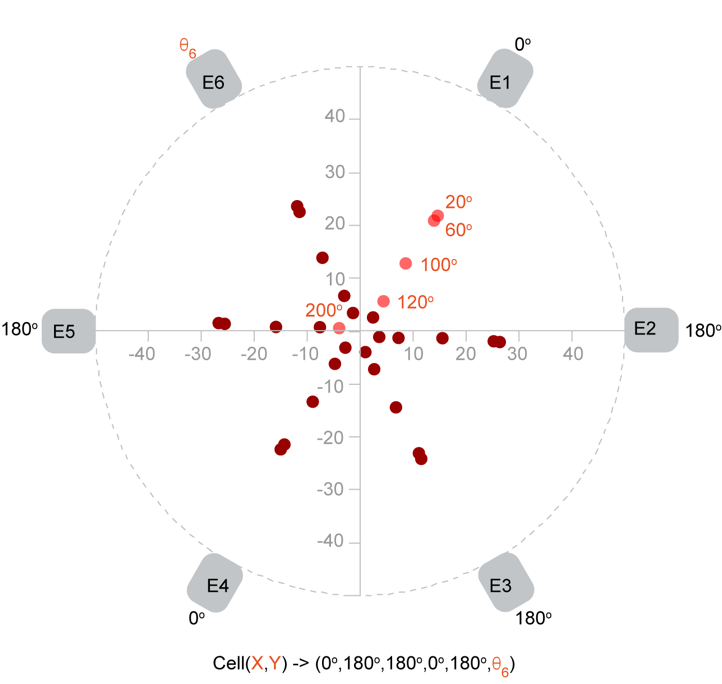


Figure S1 Cell positioning using the phase combination set to the electrodes. Here the phase of the sixth electrode (E6) is varied as indicated by the labels, and the marker shows the location in the chip. The applied phases for the other five electrodes (E1-E5) are also indicated in the plot. The dark red points are generated by rotating the experimental data points by steps of 60^o^ due to the circular symmetry of the DEP chip.


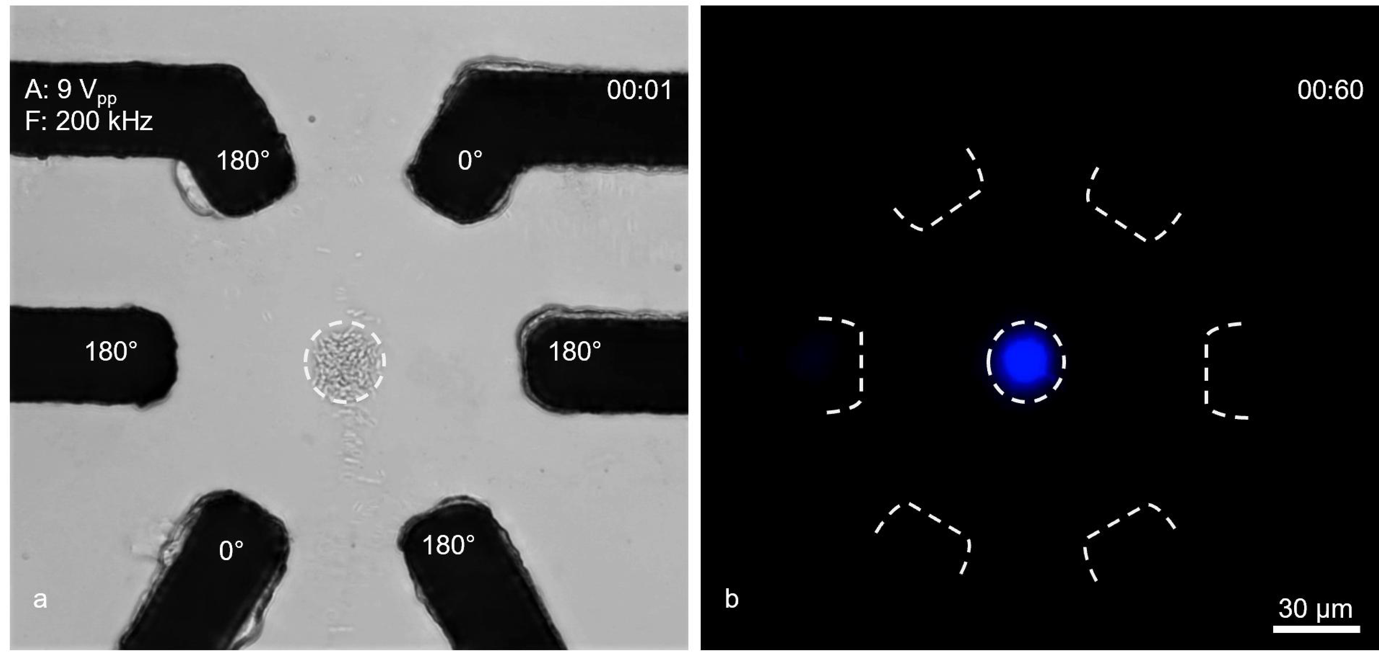


Figure S2 Fluorescence preservation on-chip by the E. coli. The (a) bright field and (b) fluorescence images show the E. coli cells trapped at the center of the imaging zone in RPMI 1640 medium for 60 minutes. In the absence of neutrophilic cells and a phagocytosis event, the fluorescence of the E. coli cells remained intact. To enhance visibility, the placement of the electrodes was marked with white dashed lines in panel (b), while the cell area was outlined with a white dashed circle.


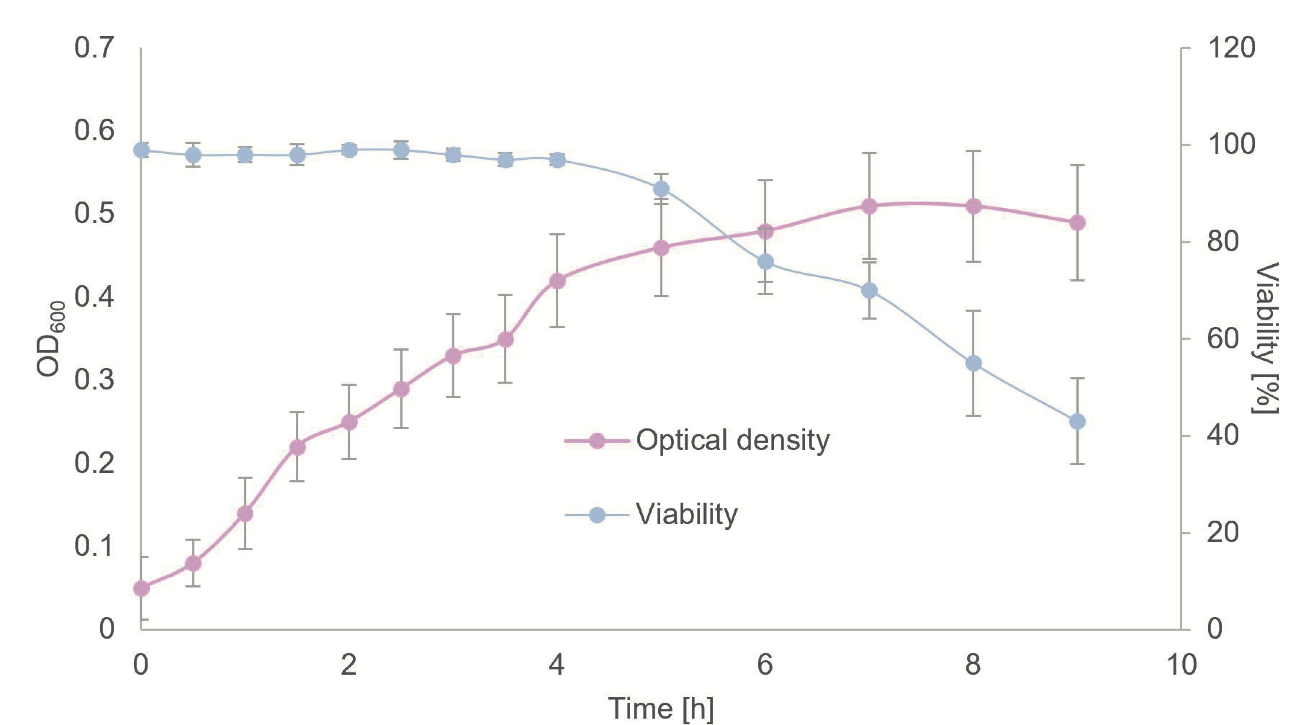


Figure S3 Growth curve and viability assessment of baker's yeast over 9 hours in DEP buffer


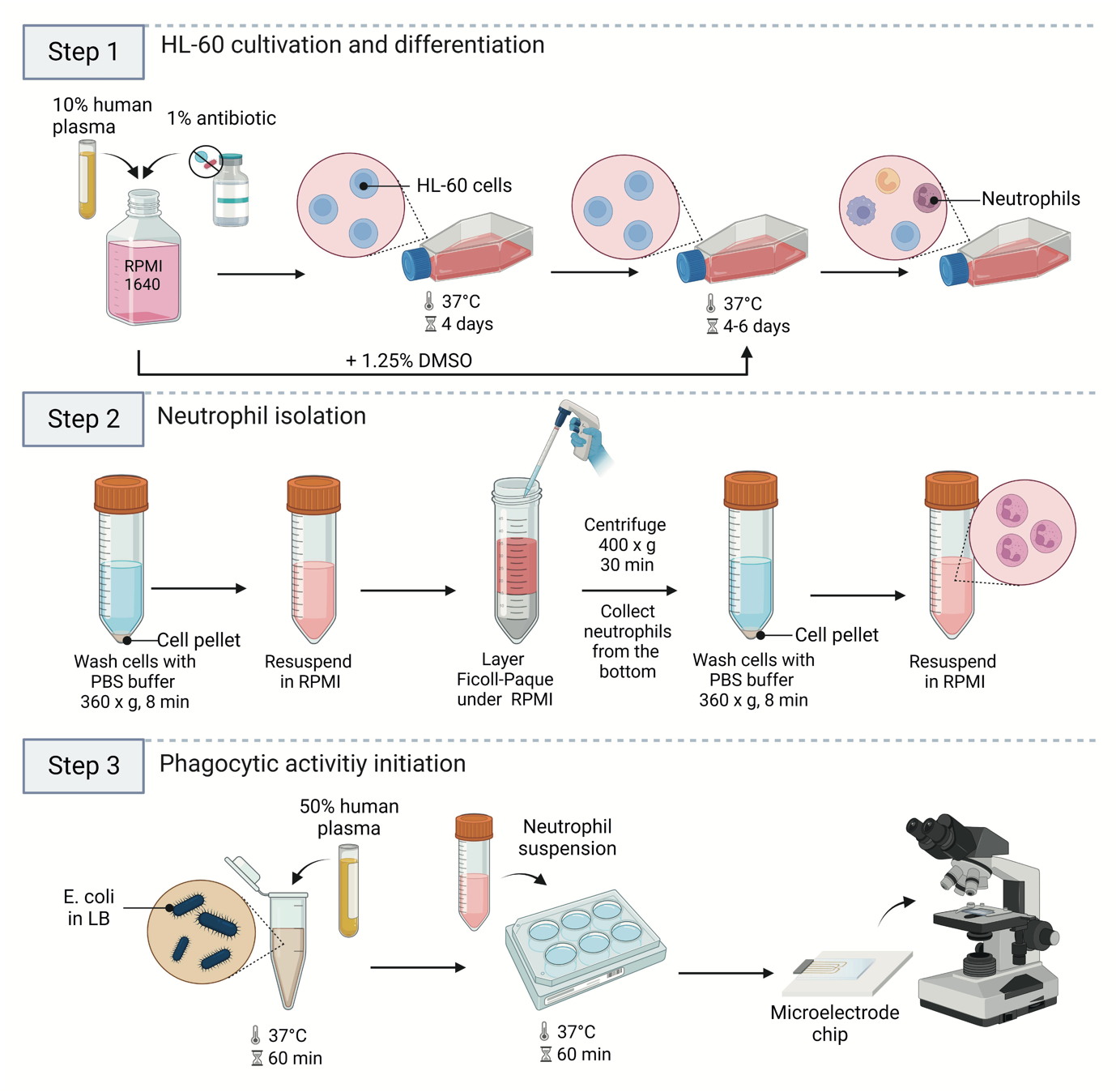


Figure S4 Cell preparation procedures for interaction experiments between HL-60 neutrophilic cells and E. coli


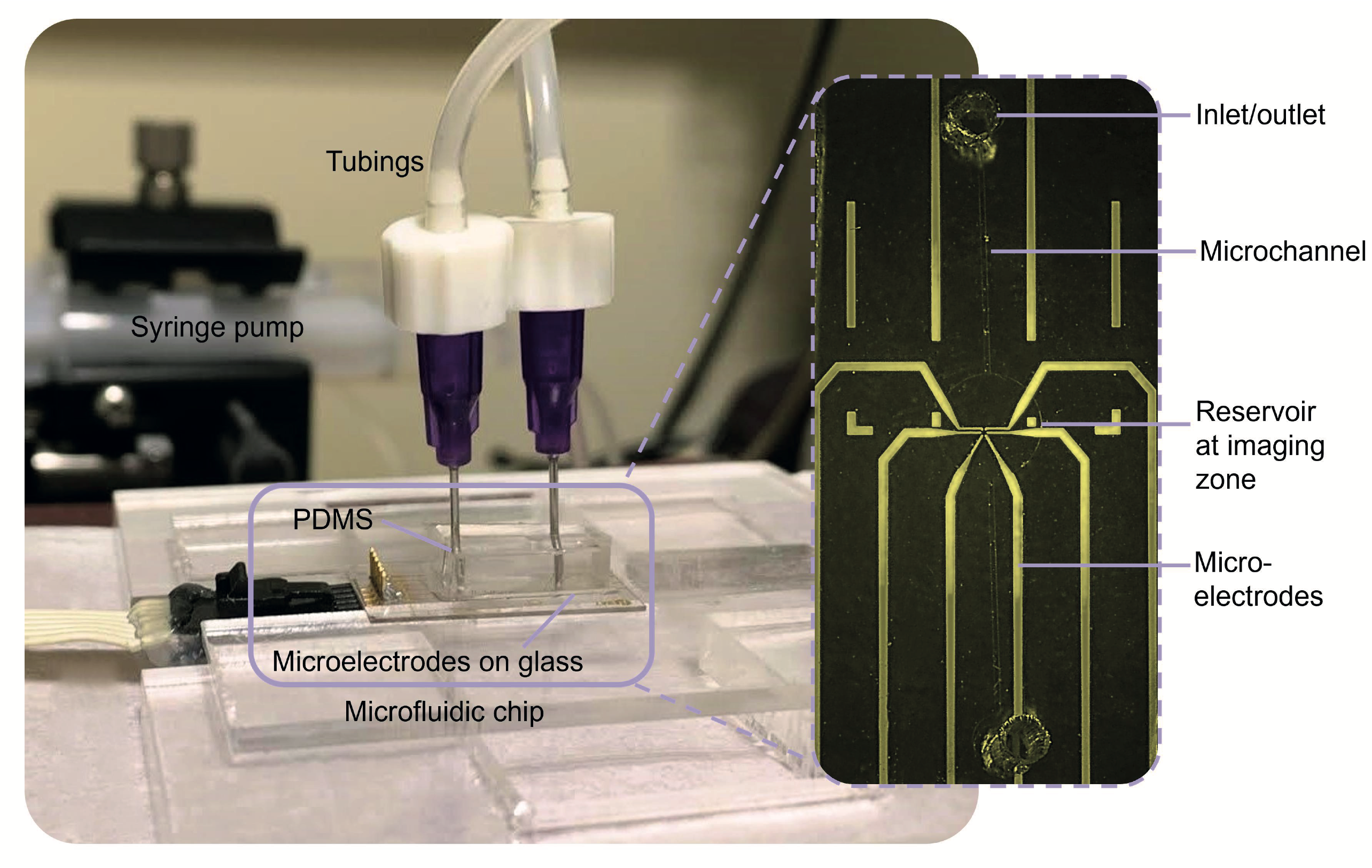


Figure S5 DEP chip with a PDMS microchannel layer

Figure S6 a-c) Frequency-dependant behaviour of B. subtilis, (d-f) dead yeast, (g-i) live yeast. The

phase values were set to (0°, 180°, 180°, 0°, 180° and 180°) in (a-c) for the B. subtilis and for the live-dead yeast (d-i) the phase was set to (0°, 90°, 180°, 0°, 90° and 180°). The caption of the colour box with the number mentions the trapping behaviour in each trap zone


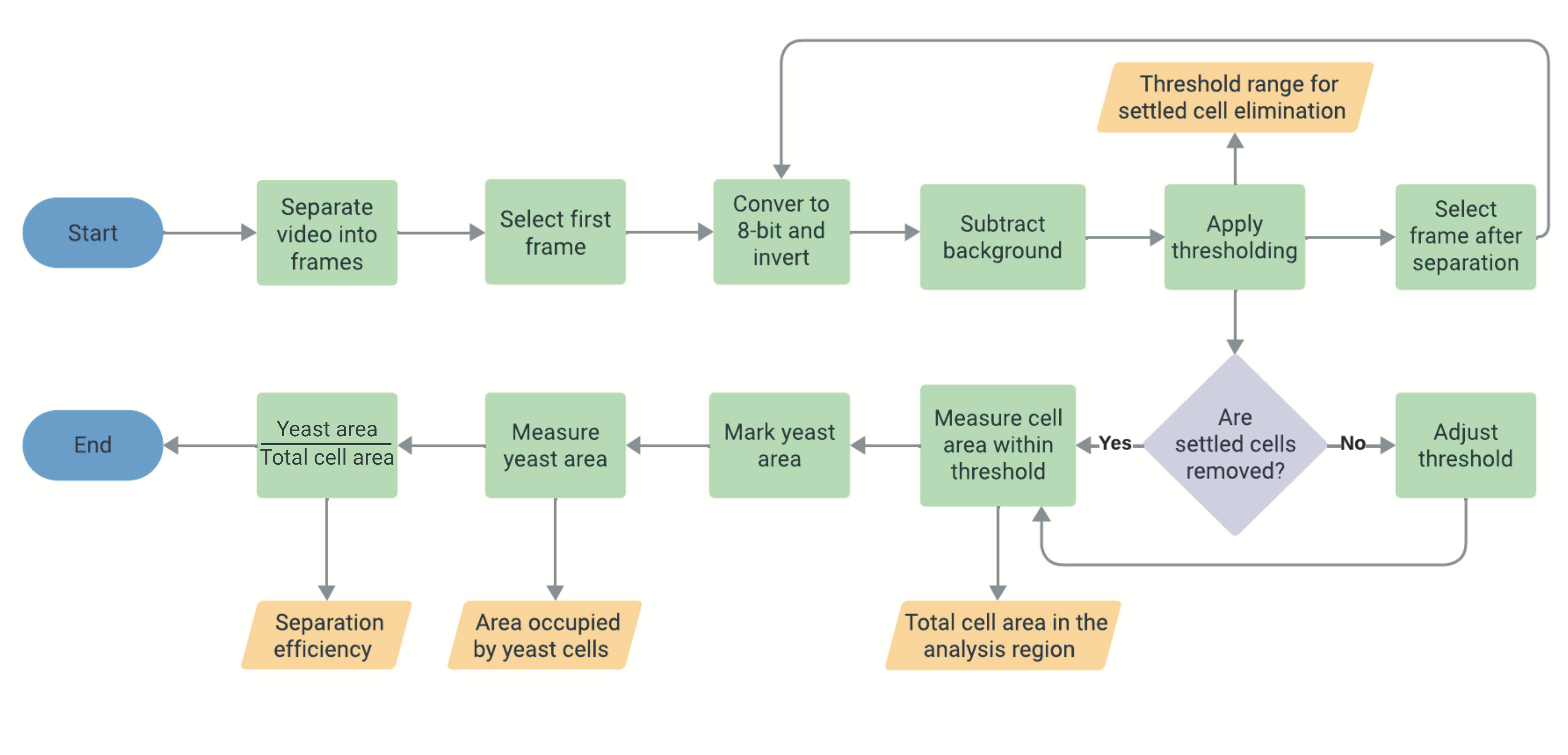


Figure S7 Separation efficiency calculation done with ImageJ
