# Supplementary material for: Portable dielectrophoresis for biology: ADEPT facilitates cell trapping, separation, and interactions
Source: Microsyst Nanoeng. 2024 Mar 1;10:29. doi: 10.1038/s41378-024-00654-z (PMC10907756; doi:10.1038/s41378-024-00654-z)
Supplement: Supplementary file 7 — Portable dielectrophoresis for biology: ADEPT [file 41378_2024_654_MOESM7_ESM.pdf]

Appendix A. Supplementary Information: Portable dielectrophoresis for biology: ADEPT facilitates cell trapping, separation, and interactions

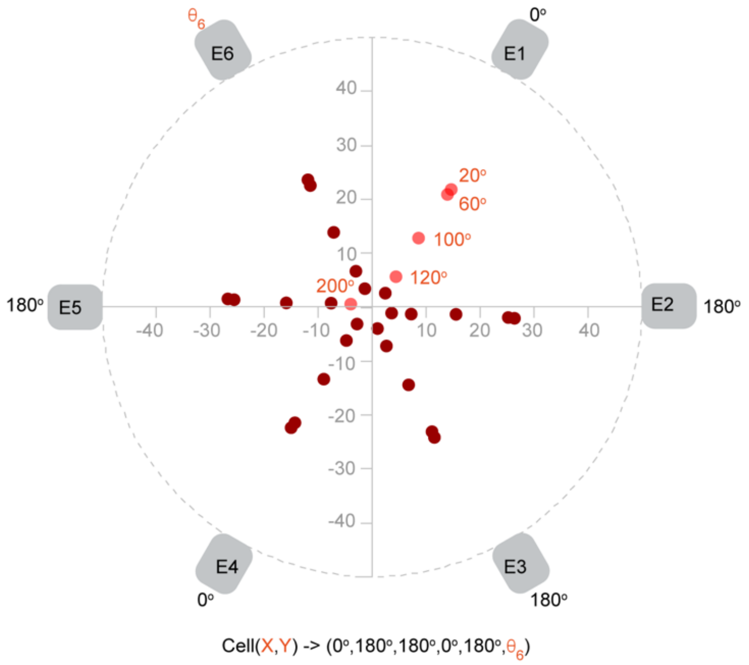

Figure A1 Cell positioning using the phase combination set to the electrodes. Here the phase of the sixth electrode (E6) is varied as indicated by the labels, and the marker shows the location in the chip. The applied phases for the other five electrodes (E1-E5) are also indicated in the plot. The dark red points are generated by rotating the experimental data points by steps of 60° due to the circular symmetry of the DEP chip.

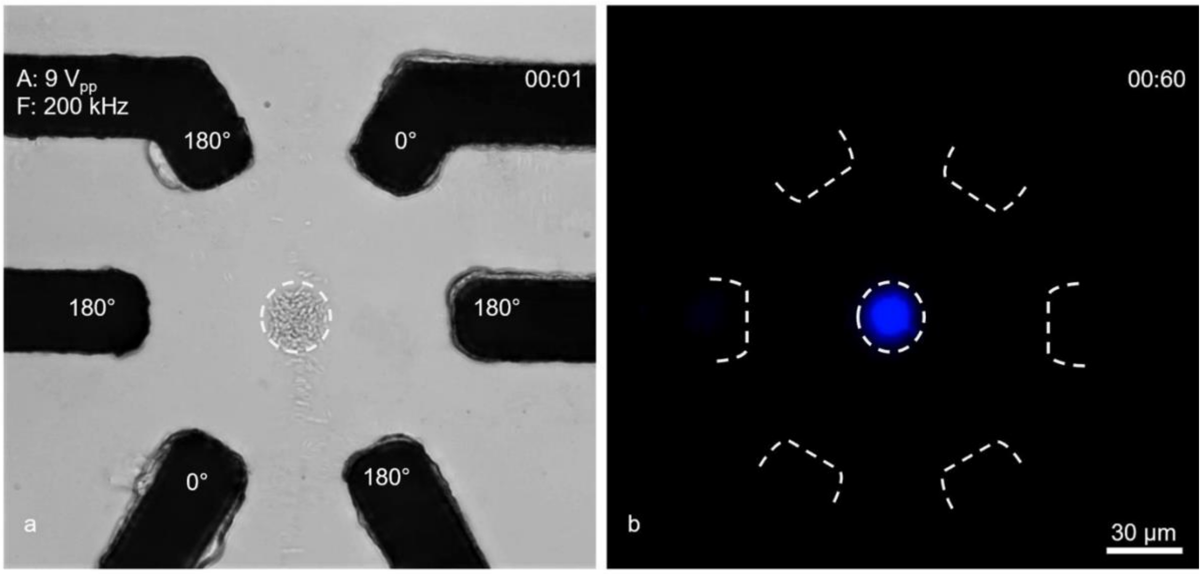

Figure A2 Fluorescence preservation on-chip by the *E. coli*. The (a) bright field and (b) fluorescence images show the *E. coli* trapped at the center of the imaging zone in RPMI 1640 medium for 60 minutes. In the absence of neutrophilic cells and a phagocytosis event, the fluorescence of the *E.* coli remained intact. To enhance visibility, the placement of the electrodes was marked with white dashed lines in panel (b), while the cell area was outlined with a white dashed circle.

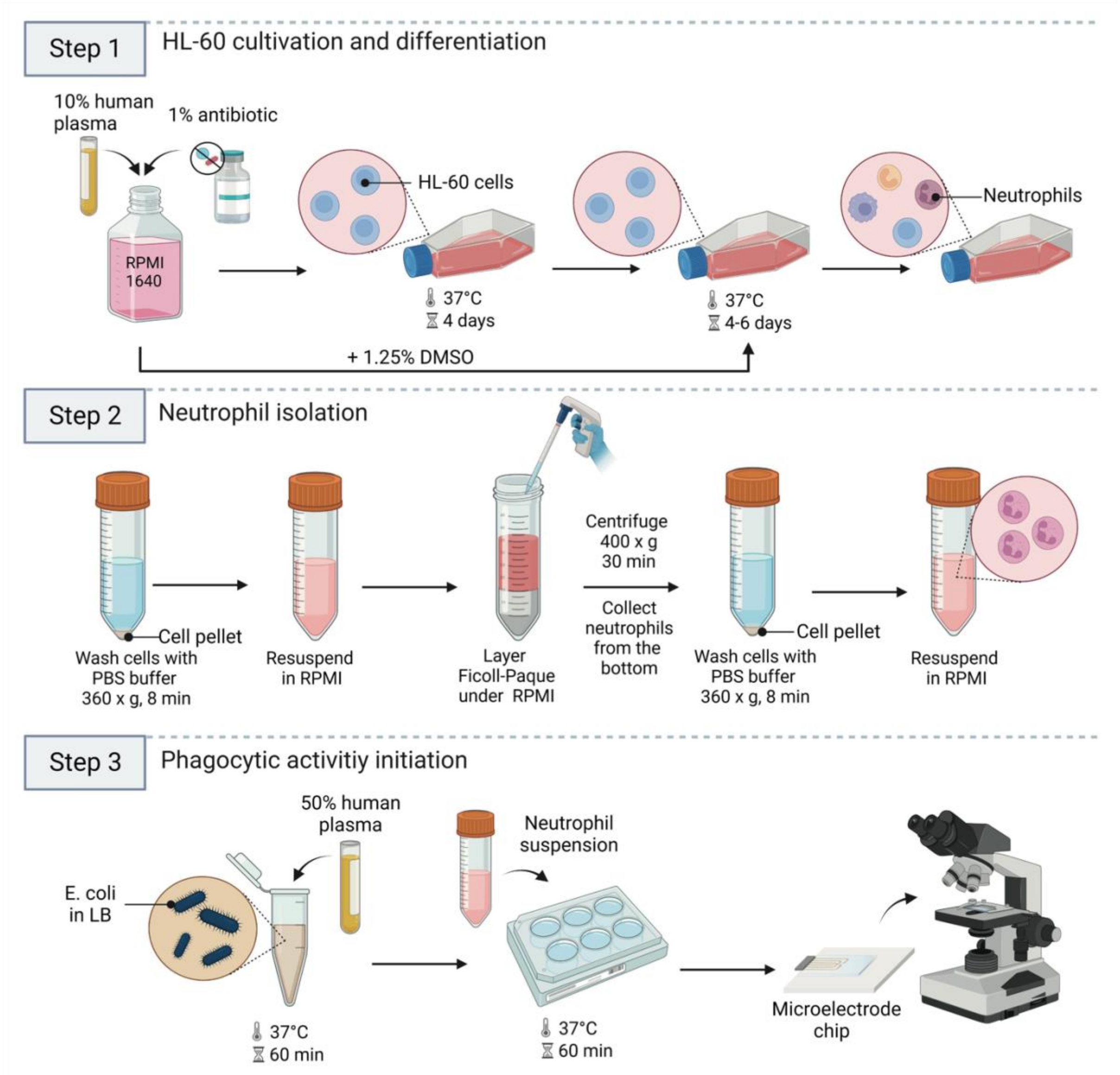

Figure A3 Cell preparation procedures for interaction experiments between HL-60 neutrophilic cells and *E. coli*
